# The Compensation Effect of Mortality: A Global Analysis of Human Populations

**Published:** 2025-11-03

**Authors:** Natalia S. Gavrilova, Leonid A. Gavrilov

**Affiliations:** NORC at the University of Chicago, 1155 E 60th Street, Chicago, IL60637, USA

**Keywords:** mortality, species-specific lifespan, longevity, life extension

## Abstract

The compensation effect of mortality (CEM) refers to the convergence of mortality rates at advanced ages across different human populations. This phenomenon occurs when higher values of the Gompertz slope parameter α (actuarial aging rate) are offset (compensated) by lower values of the intercept parameter R (initial mortality rate). The age at which this convergence occurs is known as the species-specific lifespan (SSLS). The primary aim of this study is to estimate SSLS in contemporary human populations of different world regions using both parametric and nonparametric approaches. We analyzed United Nations period abridged life tables for 251 countries and regions, spanning the period from 1980 to 2020. Both parametric and nonparametric methods of SSLS estimation produced comparable results. For industrialized countries with high-quality vital statistics, SSLS estimates ranged from 95 to 100 years—consistent with estimates made more than three decades ago. This suggests that the convergence point of CEM has remained stable over time, despite substantial declines in mortality at younger ages. High SSLS estimates were also observed in regions of Eastern and South-Eastern Asia. In contrast, other world regions showed lower SSLS values, ranging from 75 to 90 years. Due to the CEM, efforts to extend lifespan are typically accompanied by a paradoxical increase in the actuarial aging rate (Gompertz slope), making significant extensions of life expectancy at older ages challenging. The CEM thus remains a key constraint to radical life extension in humans. Importantly, the compensation effect of mortality appears to be a general regularity, observed consistently across world populations examined.

## 1. Introduction

This study explores the compensation effect of mortality (CEM)—a phenomenon characterized by mortality convergence at advanced ages. In this process, higher values of the Gompertz mortality model’s slope parameter (actuarial aging rate) are offset by lower values of the intercept (initial mortality) across different human populations [1]. The age at which this convergence occurs is referred to as the species-specific life span (SSLS) and mortality level as the species-specific mortality. As a result of CEM, factors contributing to life span extension are often paradoxically associated with an increase in the actuarial aging rate, as defined by the Gompertz slope.

In 1960, Bernard L. Strehler and Albert S. Mildvan identified an inverse relationship between the Gompertz parameters: countries with high values of the pre-exponential multiplier (R) tended to have lower values of the exponential index (α) [2]. This observation is now known as the Strehler-Mildvan (SM) correlation [3,4].

This correlation was later questioned by Gavrilov and Gavrilova (1991), who demonstrated that neglecting a non-zero Makeham term (representing background mortality) can produce a spurious Strehler-Mildvan (SM) correlation [1,5]. The concept of the “Compensation Effect of Mortality” (CEM) was first introduced in 1978, when incorporating the Makeham parameter was found to yield substantially different parameter estimates for the SM correlation [5]. CEM describes the convergence of age-dependent (senescent) mortality trajectories at advanced ages: when extrapolated using the Gompertz model, all mortality curves intersect at a single point [1,5,6]. In disadvantaged populations, higher baseline mortality rates are compensated by a slower apparent “aging rate,” reflected in a longer mortality doubling time. Because this convergence point is largely independent of environmental or genetic factors, it has been interpreted as a fundamental, species-specific characteristic of survival. For humans, this convergence occurs at approximately 95 ± 2 years [1]. The CEM phenomenon has been documented not only in humans but also in several other species [1,7,8].

**Figure 1.**
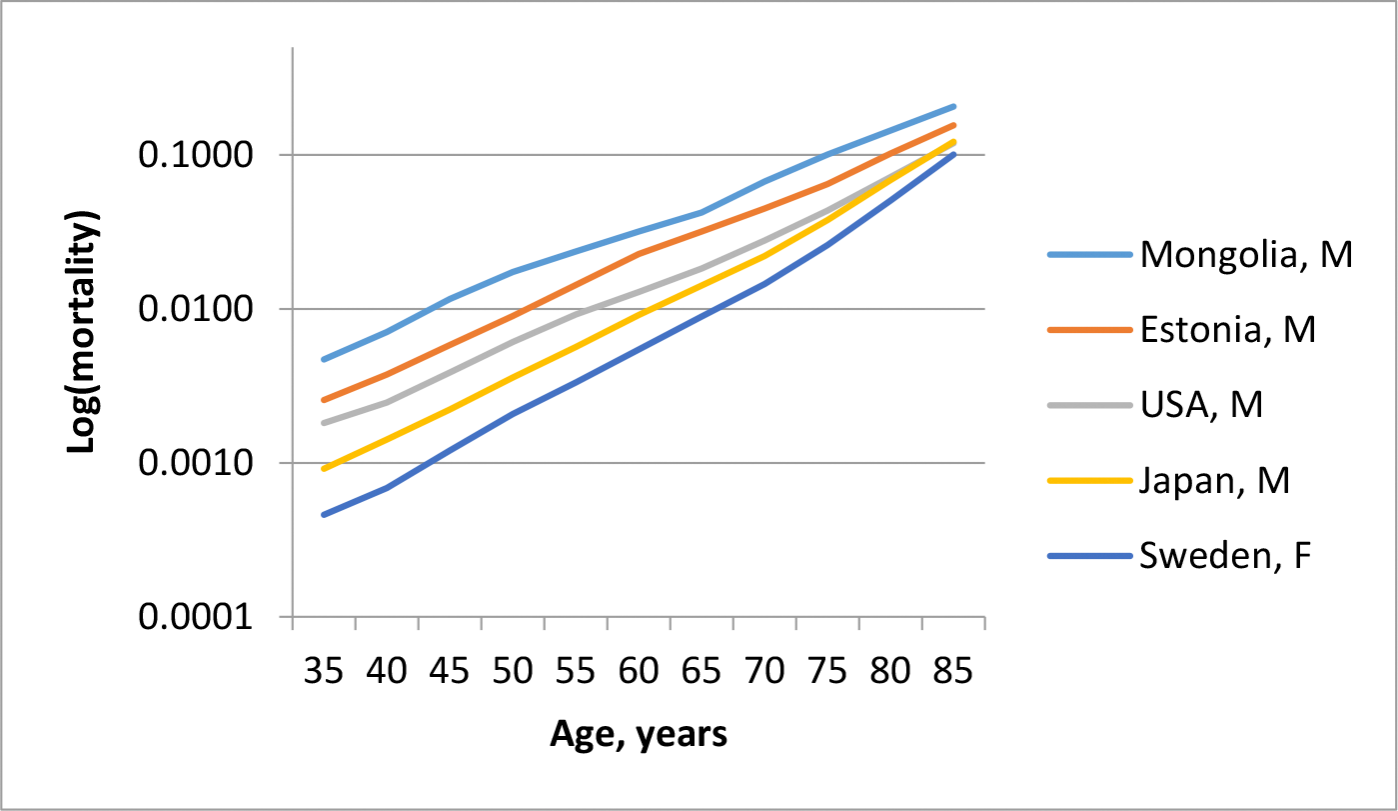
Logarithm of mortality in 2010–2015 as a function of age in five populations.

Importantly, the CEM is evident regardless of the specific parameter estimation technique. When plotting the logarithm of mortality against age for multiple populations, convergence near age 95 years consistently appears. Parameter estimation within the Gompertz framework serves only to quantify this already visible pattern. Figure 1 illustrates the Compensation Effect of Mortality (CEM) for five populations of men and women, based on period mortality data for 2010–2015. As seen in the figure, mortality variability (on a logarithmic scale) decreases at advanced ages compared to younger ages. This observation supports the prediction that as mortality convergence is approached, relative variation in mortality declines [6]. This prediction was confirmed using data from the Human Mortality Database [9]. It was found that CEM manifests as a continuous decline in relative mortality variation with age measured by both the coefficient of variation and the standard deviation of the logarithm of mortality. This decline reaches a minimum near the species-specific lifespan, estimated to be around 96–97 years [9]. This estimate closely matches lifespan values derived from correlations between Gompertz parameters (95–98 years) [1,10]. Notably, this representation of CEM is achieved through a non-parametric approach, without requiring estimation of Gompertz model parameters. Compensation effect of mortality sometimes is called a compensation law of mortality emphasizing its almost universal nature [6,10].

Despite its significance, the compensation effect of mortality and the SM correlation have received limited empirical investigation in recent decades. Most contemporary work focuses on theoretical extensions of the SM model [4,11,12]. The few empirical studies available often ignore the Makeham term, thereby replicating the same modeling issues that Gavrilov and Gavrilova had previously identified [3,4,13]. This oversight has been criticized by other scholars [14,15].

Previous studies on CEM have predominantly focused on mortality patterns in developed countries [6,10,16,17]. However, considerably less is known about whether CEM is observable in other global regions, particularly in countries across Africa and Asia. To address this gap, we conduct a large-scale analysis of life tables compiled by the United Nations, estimating the correlation between Gompertz parameters while explicitly accounting for the Makeham term (i.e., background mortality). In a complementary analysis, we examine contemporary mortality data from the period 2010–2015, under the assumption that the Makeham term becomes negligible in this timeframe and thus does not significantly influence the estimation of CEM-related parameters. Finally, we estimate species-specific lifespan across different world regions by identifying the age at which relative mortality variation is minimized. The derived CEM parameters are then systematically compared across global regions to assess the universality and variability of the compensation effect.

## 2. Materials and Methods

### 2.1. Data Sources

This study utilizes period abridged life tables from the United Nations World Population Database, which provides mortality estimates in 5-year age intervals [18]. The dataset includes mortality data for 7028 populations, separated by sex, and spans fourteen 5-year periods from 1950 to 2020. Five-year age-specific death rates were used as proxies for hazard rates. The focus of this study was on contemporary data (1980–2020, 3159 populations).

UN dataset includes life table data for multiple world regions: Europe, Sub-Saharan Africa, Northern Africa and Western Asia, Central and Southern Asia, Eastern and South-Eastern Asia, Latin America and the Caribbean, Northern America, and Australia and New Zealand. Countries of Oceania were excluded from the analysis due to their small populations, which result in less reliable mortality estimates. For many countries within these regions, mortality information is available only in the form of abridged life tables. The continental classification used in this study follows the United Nations system. We assume that the quality of vital statistics in most developing countries has significantly improved over recent decades, making mortality estimates generally reliable—particularly for working-age populations. A detailed assessment of mortality data quality in one Central Asian country (Kyrgyzstan) showed that while issues persist for infant, child, and partly for old-age mortality, statistics for working ages are reasonably accurate [19,20].

### 2.2. Statistical Analysis

In the first stage of analysis, we estimated parameters R and α (intercept and slope) of the Gompertz-Makeham model using the following hazard rate equation:


(1) $$\mu_{x}=A+R\;\exp(\alpha x)$$

where:

- $\mu_{x}$ is the hazard rate at age x,
- A is the Makeham parameter (age-independent or background mortality),
- R is the pre-exponential multiplier or Gompertz intercept,
- α is the Gompertz slope parameter or actuarial aging rate.

Parameter estimation was performed using non-linear regression, over the age interval 30–85 years. Non-linear regression model is often used in demography and biology for parameter estimation of non-linear dependencies [21–23]. The lower age threshold of 30 years avoids the confounding effects of early adult mortality (e.g., the “accident hump”), while the upper threshold of 85 years excludes the influence of mortality deceleration at advanced ages. We evaluated standard Gompertz-Makeham model and constrained Gompertz-Makeham model with non-negative Makeham term (background mortality). The constrained model keeps the Makeham parameter A to be non-negative, reflecting its interpretation as background mortality. This avoids biologically implausible negative values. It was found that both models produce similar results for parameters R and α, so we used results from the constrained model for further analyses.

Nonlinear regression is not the only approach to estimate the parameters of the Gompertz-Makeham model. An alternative method, proposed earlier [10], relies on linear regression of log-transformed mortality data. In this study, we applied a similar approach for estimating Gompertz parameters using linear regression of log-transformed mortality [3]. This method is based on the observation that, in contemporary populations, the Makeham term has become negligible and can therefore be omitted. This assumption is supported by previous findings showing that species-specific lifespan (SSLS) estimated using the Gompertz-Makeham and Gompertz models is virtually identical in contemporary populations [16]. Consequently, accounting for the Makeham term does not materially affect the parameters describing the Compensation Effect of Mortality (CEM). For this reason, we performed an additional set of analyses using linear regression of log-transformed mortality in 2010–2015 for the age range 30–95 years-a broader interval that includes older ages where the contribution of Makeham term is minimal.

After estimating the Gompertz parameters for each major world region, we then conducted a linear regression of the following form:


(2) ln(R)=ln(M)−Bα

where:

- B is the species-specific life span (SSLS), estimated as the negative slope of the regression,
- ln(M) is the regression intercept or the logarithm of species-specific mortality.

This regression framework enables us to test the validity of the compensation effect of mortality using contemporary demographic data. As for the regression diagnostics, the normality of residuals was acceptable for all six regions. Heteroscedasticity of residuals was not detected for two world regions with the highest SSLS and reasonably good quality of vital statistics (industrialized countries and the countries of Eastern and South-Eastern Asia).

In addition to the two regression models used for estimation of the Gompertz parameters we applied a non-parametric method of SSLS estimation, which does not assume any specific form of mortality trajectories. Non-parametric estimation of the species-specific lifespan was done by calculating the age of minimal value for the coefficient of variation of mortality. Coefficient of variation (which is the ratio of the standard deviation to the mean) for mortality was calculated as a function of age using age-specific death rates data in 2010–2015 from corresponding life tables available for each region. All calculations were conducted using Stata statistical software, release 16 (procedures *summarize*, *nlin* and *regress*).

## 3. Results

### 3.1. Compensation Effect of Mortality in Different Regions of the World

Table 1 presents the results of linear regression analysis between the logarithm of R and α (as specified in Equation (2)), based on the constrained Gompertz-Makeham model. The study was done for several large regions as listed in the UN database. Among these, industrialized countries of Europe, Northern America, Australia and New Zealand—which have the highest quality of demographic data—also exhibit one of the highest estimate of species-specific lifespan, calculated as 96.2 ± 0.3 years. This estimate is similar for men (95.5 ± 0.5 years) and women (95.9 ± 0.7 years). It also closely aligns with earlier findings reported more than thirty years ago (95 ± 3 years) [1].

Countries of Eastern and South-Eastern Asia also demonstrate very high SSLS—96.7 ± 2.0 years for men and 99.6 ± 1.6 years for women. These countries on average have lower mortality and higher life expectancy. Thus, a clear pattern is observed: as the quality of data declines, the estimated SSLS also tends to decrease. However, an alternative explanation is possible-countries with lower data quality may also experience less favorable living conditions, which could contribute to accelerated aging.

Table 2 presents the CEM parameters for six world regions estimated using an alternative method for calculating Gompertz parameters. The results show strong consistency with those obtained from the nonlinear regression approach. Once again, low-mortality countries, along with those in Eastern and South-Eastern Asia, exhibit the highest SSLS values. Women from Eastern and South-Eastern Asia, in particular, show exceptionally high SSLS levels. In contrast, other regions display substantially lower SSLS values. Interestingly, Sub-Saharan Africa demonstrates higher SSLS values than might be expected given its very low life expectancy, whereas Latin America and the Caribbean show lower-than-expected SSLS estimates.

### 3.2. Non-Parametric Estimates of the Species-Specific Lifespan

Table 3 shows non-parametric estimates of SSLS using coefficient of variation for mortality. In this case SSLS was estimated as an age of minimal coefficient of mortality variation. Note that these estimates of SSLS are mostly in agreement of estimates obtained using the Gompertz parameters. The highest values of SSLS are observed for industrialized countries, Eastern and South-Eastern Asia and female population of Latin America and the Caribbean. On average, regions exhibiting higher life expectancy also show higher values of SSLS. There are some anomalies, however. For example, Eastern and South-Eastern Asia and Latin America and the Caribbean have similar values of life expectancy, but very different levels of SSLS. Sub-Saharan Africa shows very low life expectancy while its level of SSLS is average compared to other regions.

Overall, these results demonstrate that CEM parameters and SSLS in particular may differ in different regions of the world although there is a consistency in SSLS estimates across different methods of their estimation.

## 4. Discussion and Conclusions

In this study, we analyzed CEM and estimated its parameters using both parametric and non-parametric methods with period abridged life tables from the United Nations World Population Database, covering 251 countries and regions across 8 five-year time periods (1980–2020).

The Gompertz parameters (R and α) needed for CEM parameters calculation were estimated using two methods. One method used non-linear regression with non-negative Makeham term over the age range 30–85 years. Another method was based on a linear regression of log-transformed mortality rates over wider age range 30–95 years assuming negligible Makeham parameter for contemporary populations. We then performed linear regression with ln(R) as the dependent variable and α as the independent variable. The slope of this regression (denoted as B) represents the species-specific lifespan (SSLS), as it corresponds to the age at which mortality trajectories intersect on a semi-log scale after extrapolation [1]. Results were mostly consistent for all three methods of SSLS estimation. Among all country groups, industrialized countries—with the most reliable demographic data—showed one of the highest SSLS, estimated at 96.2 ± 0.3 years and 98.9± 2.5 years, closely matching the earlier estimate of 95 ± 3 years made by more than thirty years ago [1]. These estimates are close to other published estimates of SSLS based on the Human Mortality Database data: 98.0 ± 1.9 for men and 97.2 ± 0.7 for women [10].

Among the world regions examined, Eastern and South-Eastern Asia showed remarkably high SSLS values, especially among women. Notably, one East Asian population—Japan—exhibits unusually low actuarial aging rates relative to its low Gompertz intercept [9]. As a result, Japanese women experience exceptionally low mortality at advanced ages [9], deviating from typical CEM trajectories.

Other world regions display lower SSLS values compared to industrialized countries, which may reflect either lower data quality in vital statistics or less favorable living conditions that contribute to accelerated aging. Overall, these findings indicate that the parameters of CEM can vary substantially across different world regions.

Nonparametric estimates of SSLS were generally higher than those obtained from correlations between Gompertz parameters. Similar to previous estimation methods, the nonparametric approach revealed high SSLS values for industrialized countries and for nations in Eastern and South-Eastern Asia. Because abridged life tables provide limited age detail, nonparametric SSLS estimates derived from them are less precise. Nevertheless, earlier nonparametric analyses based on Human Mortality Database complete life tables estimated SSLS at approximately 96 years for both sexes [9], which closely aligns with the results obtained in the present study.

Estimates of the CEM in industrialized countries with low mortality demonstrate remarkable stability over time [1,9,16]. This stability indicates that the CEM convergence point remains consistent despite decades of substantial mortality decline at younger ages. Further research has shown that the CEM pattern persists across populations with different levels of familial longevity including siblings of both centenarians and short-lived individuals [24]. These findings suggest that familial longevity and related genetic factors exert little influence on survival beyond age 100, near the SSLS range. Mortality crossovers were observed not only for persons with different levels of familial longevity but also for persons with different income [25], for different racial and ethnic groups [26,27] and different regions [28].

Recently, new developments have expanded the application of the CEM framework. Milevsky (2020) proposed using CEM to derive a variant of biological age termed the longevity-risk-adjusted global age [10]. In this approach, the mortality trajectory of a given population is compared with global Gompertz-Makeham parameters, estimated as a weighted average of local parameter values. Within this framework, populations with steeper Gompertz slopes correspond to lower biological ages, and vice versa. Specific biological age values for each chronological age can be computed using a defined formula [10]. This method was later applied to analyze regional mortality patterns in Italy and Russia [17]. The authors observed correlations between the Gompertz parameters after accounting for the Makeham term and estimated corresponding regional longevity-risk-adjusted global ages.

The two studies mentioned earlier [10,17] assumed that mortality remains constant beyond the SSLS age. However, real-world data show that mortality after age 100 is not constant but instead continues to increase, albeit at a slower rate—a phenomenon known as mortality deceleration. In fact, mortality patterns after age 90 in period data have not yet been examined in sufficient detail. For cohort data, however, more extensive research exists. In some countries, particularly the United States, cohort mortality has been shown to follow the Gompertz law up to ages 105–106 years [29]. By contrast, in period data, mortality deceleration at advanced ages appears more common. For cohort data, it has been shown that mortality trajectories indeed cross over around ages 97–100 years [29] when comparing U.S. older and more recent birth cohorts. For period data, additional research is needed to determine whether a similar pattern occurs. Further investigation of period mortality beyond the SSLS age represents a promising direction for future research.

Some researchers have argued that the Strehler–Mildvan correlation arises from statistical interdependence between the Gompertz parameters, implying that both this correlation and the CEM may be statistical artifacts [30]. Later studies controlling for data collinearity showed that statistical interdependence of parameters makes very small contribution to overall variation [16]. Figure 1 demonstrates that it is not necessary to estimate Gompertz parameters to establish the presence of CEM, thereby rendering such critiques less relevant. Another source of spurious correlation arises when working with small sample sizes. Simulation studies have shown that traditional statistical methods tend to underestimate the Gompertz slope and overestimate the intercept under such conditions. Nonetheless, these issues are generally not a concern in human demographic research, where large sample sizes are more common [31]. These considerations highlight the advantages of using non-parametric approaches to study CEM, as they avoid potential biases associated with parameter estimation.

One theoretical explanation for the compensation effect of mortality (CEM) comes from the reliability theory of aging [1,32]. According to this framework, the rate of underlying destructive processes in aging may be a species-specific invariant, independent of environmental or genetic factors. This should not be confused with theories of programmed death. Rather, it reflects the robustness of biological systems, where many vital parameters remain highly stable—such as body temperature (~36.7°C), which shows little variation by sex, ethnicity, or environment. Some processes governed by such stable parameters, like racemization of L-amino acids, are even used to estimate biological age [33].

Estimates of the SSLS may be of interest to aging research, as they offer insights into the rate at which vital physiological components are lost due to aging—often referred to as the true aging rate. According to reliability models of aging [1,34], this rate is approximately the inverse of the species-specific lifespan (1/B, where B is derived from Equation (2)). Empirical studies have shown that SSLS in humans remains relatively stable over time, typically ranging from 95 to 98 years [1,10,16]. This implies an approximate annual loss of vital elements at a rate of 1%. Interestingly, this estimated rate closely matches observed rates of annual cellular loss in various neural tissues, which range from 0.6% to 1.6% per year [35,36]. It also is close to the telomere shortening rate in humans—0.5% per year [37]. These estimates were made for countries with higher life expectancy and better quality of vital statistics. In the current study we found that in many regions of the World SSLS is lower than 95 years and ranges from 72 to 90 years. It is possible that populations in these regions live in less optimal conditions and hence their “true” biological aging rate is somewhat higher than 1% and varies from 1.1 to 1.3% per year. Alternatively, it can be related to lower quality of mortality statistics in these regions.

A key implication of CEM is that in humanslife span extension is often accompanied by a paradoxical increase in the actuarial aging rate (the Gompertz slope), making significant extensions of life expectancy at older ages challenging. Thus, CEM presents a major obstacle to further extending life span in humans. Animal studies have shown that substantial life extension is achieved primarily by *reducing*—rather than *increasing*-the Gompertz slope [38]. Therefore, to identify human populations with slower aging, it is important to focus on those exhibiting both low initial mortality and a low actuarial aging rate. Exploring the lifestyle and genetic factors characteristic of such populations may provide valuable insights and promising directions for future longevity research.

## Figures and Tables

**Table 1.** Characteristics of CEM based on the constrained Gompertz-Makeham model for period 1980–2020. United Nations database *.

	Regression Coefficients		Correlation Coefficient between ln(R) and α	Number of Life Tables

Population	ln(M) ± σ	B ± σ, Years		

Countries of Europe, Northern America, Australia and New Zealand				
Men	−0.76 ± 0.05	95.51± 0.53	−0.9937	408
Women	−0.74 ±0.08	95.93 ±0.71	−0.9891	408
Total	−0.76 ±0.05	96.22± 0.34	−0.9947	816
Sub-Saharan Africa				
Men	−2.51±0.10	72.31 ±1.05	−0.9570	440
Women	−1.46± 0.09	85.03± 0.88	−0.9771	439
Total	−1.49 ± 0.07	84.05 ±0.67	−0.9729	879
Northern Africa and Western Asia				
Men	−2.11± 0.13	79.29±1.40	−0.9671	224
Women	−2.15± 0.13	81.00 ± 1.33	−0.9712	224
Total	−1.81± 0.09	83.51 ±0.98	−0.9707	448
Central and Southern Asia				
Men	−2.36± 0.16	75.71± 1.88	−0.9612	136
Women	−2.04± 0.13	81.40 ±1.47	−0.9789	136
Total	−1.91±0.10	82.10± 1.17	−0.9737	272
Eastern and South-Eastern Asia				
Men	−0.61 ±0.18	96.69 ±1.98	−0.9654	176
Women	−0.35 ±0.17	99.63 ±1.61	−0.9782	176
Total	−0.42 ±0.10	98.86 ± 1.06	−0.9805	352
Latin America and the Caribbean				
Men	−2.10 ± 0.11	82.27 ± 1.32	−0.9595	336
Women	−2.05 ±0.14	84.75 ±1.35	−0.9610	328
Total	−1.69 ±0.08	87.64± 0.86	−0.9697	664

* Gompertz parameters are estimated using non-linear regression model.

**Table 2.** Characteristics of CEM based on the Gompertz model for period 2010–2015. United Nations database *.

	Regression Coefficients		Correlation Coefficient between ln(R) and α	Number of Life Tables

Population	Ln(M) ± σ	B ± σ, Years		

Countries of Europe, Northern America, Australia and New Zealand				
Men	−1.12 ± 0.27	94.26 ± 2.97	−0.9765	51
Women	−2.11 ± 0.53	86.70 ± 5.25	−0.9207	51
Total	−0.78 ± 0.24	98.95 ± 2.53	−0.9689	102
Sub-Saharan Africa				
Men	−1.16 ± 0.24	89.64 ± 3.27	−0.9666	55
Women	−1.41 ± 0.25	88.83 ± 3.27	−0.9659	55
Total	−1.21 ± 0.18	90.26 ± 2.47	−0.9618	110
Northern Africa and Western Asia				
Men	−2.11 ± 0.13	79.29 ± 1.40	−0.9671	28
Women	−2.15 ± 0.13	81.00 ± 1.33	−0.9712	28
Total	−1.47 ± 0.29	89.05 ± 2.99	−0.9710	56
Central and Southern Asia				
Men	−2.01 ± 0.54	81.24 ± 6.42	−0.9562	17
Women	−2.39 ± 0.36	79.19 ± 3.95	−0.9818	17
Total	−1.89 ± 0.31	83.81 ± 3.51	−0.9731	34
Eastern and South-Eastern Asia				
Men	−1.34 ± 0.55	91.13 ± 6.30	−0.9554	22
Women	−0.41 ± 0.84	112.39 ± 8.93	−0.9423	22
Total	−0.35 ± 0.46	103.55 ± 5.09	−0.9528	44
Latin America and the Caribbean				
Men	−2.93 ± 0.20	74.09 ± 2.57	−0.9768	42
Women	−2.42 ± 0.35	83.52 ± 4.02	−0.9567	42
Total	−2.26 ± 0.20	84.06 ± 2.41	−0.9679	84

* Gompertz parameters are estimated using linear regression of log-transformed mortality.

**Table 3.** Non-parametric estimates of the SSLS for regions of the world.

Region	Men		Women	

	SSLS *	e_0_ in 2010–2015 **	SSLS *	e_0_ in 2010–2015 **

All countries	95	68.53	95	73.31
Industrialized countries (Europe, Northern America, Australia and New Zealand)	100		100	
Sub-Saharan Africa	75	56.20	85	59.50
Northern Africa and Western Asia	80	70.09	80	74.67
Central and Southern Asia	75	66.80	70	69.38
Eastern and South-Eastern Asia	100	72.33	100	77.61
Latin America and the Caribbean	80	71.20	100	77.72
Europe	100	73.60	100	80.72

* Species-specific lifespan defined as the age at minimal mortality variation (minimal coefficient of variation for mortality) in 2010–2015.

** Life expectancy at birth in 2010–2015.

## Data Availability

The data presented in this study are available at the following URL: https://population.un.org/wpp/downloads?folder=Standard%20Projections&group=Mortality.

## Abbreviations

CEM: Compensation effect of mortality
SSLS: Species-specific life span
